# Specific Deficit in Implicit Motor Sequence Learning following Spinal Cord Injury

**DOI:** 10.1371/journal.pone.0158396

**Published:** 2016-06-29

**Authors:** Ayala Bloch, Dror Tamir, Eli Vakil, Gabi Zeilig

**Affiliations:** 1 The National Institute for the Rehabilitation of the Brain Injured, Tel Aviv, Israel; 2 Department of Psychology, The Hebrew University of Jerusalem, Jerusalem, Israel; 3 Leslie and Susan Gonda (Goldschmied) Multidisciplinary Brain Research Center, Bar-Ilan University, Ramat-Gan, Israel; 4 Department of Psychology, Bar-Ilan University, Ramat-Gan, Israel; 5 Department of Neurological Rehabilitation, Chaim Sheba Medical Center, Tel Hashomer, Israel; 6 Sackler Faculty of Medicine, Tel Aviv University, Tel Aviv, Israel; University of Toronto, CANADA

## Abstract

**Background:**

Physical and psychosocial rehabilitation following spinal cord injury (SCI) leans heavily on learning and practicing new skills. However, despite research relating motor sequence learning to spinal cord activity and clinical observations of impeded skill-learning after SCI, implicit procedural learning following spinal cord damage has not been examined.

**Objective:**

To test the hypothesis that spinal cord injury (SCI) in the absence of concomitant brain injury is associated with a specific implicit motor sequence learning deficit that cannot be explained by depression or impairments in other cognitive measures.

**Methods:**

Ten participants with SCI in T1-T11, unharmed upper limb motor and sensory functioning, and no concomitant brain injury were compared to ten matched control participants on measures derived from the serial reaction time (SRT) task, which was used to assess implicit motor sequence learning. Explicit generation of the SRT sequence, depression, and additional measures of learning, memory, and intelligence were included to explore the source and specificity of potential learning deficits.

**Results:**

There was no between-group difference in baseline reaction time, indicating that potential differences between the learning curves of the two groups could not be attributed to an overall reduction in response speed in the SCI group. Unlike controls, the SCI group showed no decline in reaction time over the first six blocks of the SRT task and no advantage for the initially presented sequence over the novel interference sequence. Meanwhile, no group differences were found in explicit learning, depression, or any additional cognitive measures.

**Conclusions:**

The dissociation between impaired implicit learning and intact declarative memory represents novel empirical evidence of a specific implicit procedural learning deficit following SCI, with broad implications for rehabilitation and adjustment.

## Introduction

After an acute care phase, individuals with spinal cord injury (SCI) often face an intensive process of physical and psychosocial rehabilitation. As this process leans heavily on learning and practicing new skills, compromised cognitive abilities are likely to affect it detrimentally [1]. Indeed, recent work has shown that higher cognitive capacity is associated with improved adjustment in adults with SCI [2].

At 40–50%, the reported incidence of cognitive impairment following SCI is high [3]. However, such deficits are frequently attributed to concomitant brain injury [3–7] or to premorbid conditions such as poor intellectual or occupational functioning [8,9], previous brain injuries, alcoholism or drug abuse [3], low blood pressure [10], and psychiatric disorders. The few studies exploring cognitive functioning following SCI in the absence of the aforementioned conditions report deficits in processing speed, learning, memory, and attention [1,5], though a consistent profile has not been established. When concomitant head injury is ruled out, one prominent explanation for such deficits involves secondary changes to brain organization or activity resulting from SCI [11]. Reactive depression has also been highlighted as a possible source of cognitive decline, based on its elevated incidence in SCI [12–14] and negative influence on cognitive performance [15]. Finally, recent research showing changes in spinal excitability [16] and plasticity [17] during learning supports the possibility that decline in certain cognitive functions is linked to spinal cord damage directly.

In expanding the study of cognitive deficits in SCI, it is vital to examine skills that directly affect the specific obstacles encountered during rehabilitation. In this context, implicit procedural learning, or the development of routine skills without reliance on conscious or explicit memory processes [18], appears to be particularly relevant. Procedural learning is an important requisite in the rehabilitation process [9], as individuals with SCI must learn to perform daily activities under new circumstances. However, despite research relating motor sequence learning to changes in spinal cord activity [16,17], implicit procedural learning following spinal cord damage has not been examined. Based on broad clinical observations suggesting that new skill learning is often impeded among individuals with SCI, with important implications for rehabilitation, this novel examination is highly warranted.

Thus, the aim of the current study was to determine whether SCI resulting in paraplegia and not involving concomitant brain injury was associated with a specific deficit in implicit motor sequence learning. To this end, the serial reaction time (SRT) task [19] was employed, which is commonly used to study learning- and memory-related behaviors, most notably implicit learning of a motor skill [20]. To assess implicit learning, the task enables comparison of response times to a repeated sequence of stimuli (which presumably elicits implicit learning) and a novel sequence. When procedural memory is intact, reaction times tend to decrease gradually over the course of the original predefined sequence blocks, which is taken to indicate implicit procedural learning, and then increase sharply during the unlearned block [20]. Several studies have shown that this pattern is not evident or less pronounced in participant groups with various forms of damage to the procedural memory system [21].

To address potential confounding factors, additional cognitive functions including explicit learning and general intelligence were assessed in the current study, as was depression. Based on the aforementioned literature and clinical observations, it was hypothesized that the SCI group would show inferior learning on the SRT task, as compared to healthy controls, and that this deficit would not be explained by depression or by impairments in other cognitive measures.

## Materials and Methods

### Participants

Eighteen individuals with SCI resulting in paraplegia were recruited. All had acquired SCI in T1-T11, graded as American Spinal Injury Association (ASIA) Impairment Scale (AIS) A or B [22], and unharmed upper limb motor and sensory functioning according to medical records. Participants entered the study during or immediately after rehabilitation at the Chaim Sheba Medical Center Department of Neurological Rehabilitation, less than two years following injury. Two measures were employed to define absence of concomitant brain injury among participants with traumatic spinal cord injuries: 1. Absence of post-traumatic amnesia (PTA) [23] and loss of consciousness post-injury (Glasgow Coma Scale (GCS) [24] rating 14/15 or 15/15); 2. Absence of neuroimaging findings indicating brain injury in six patients with traumatic, non-penetrating, spinal cord injury; others were not subjected to imaging as there was no clinical indication to do so. Additional exclusion criteria included: alcohol or drug abuse, history of learning disability, dementia, prior brain/spinal cord surgery, and other conditions (e.g., epilepsy, movement disorders) known to affect the central nervous system. Based on these criteria, 8 participants were excluded. The remaining 10 participants (one female) had a mean age of 38.1 years (range: 21–57) and a median time from injury of 4 months (range: 2–18). Patients received various medications in accordance with their personal treatment plans, previous to and during participation in the study. Clinical and demographic information for the experimental group is detailed in Table 1.

**Table 1:** Spinal cord injury group: Demographic and clinical information.

Parti-cipant	Sex	Age (years)	Education	Dominant hand	Cause	AIS	LOI	Months since injury
1	M	38	High school	R	Bicycle accident	A	T5	2
2	M	21	University	R	Car accident	B	T3	5
3	M	38	University	R	Bicycle accident	A	T7	4
4	M	47	University	L	Spinal abscess	B	T1	6
5	M	22	High school	R	Fall	A	T4	3
6	M	28	High school	L	Gunshot wound	B	T12	4
7	M	24	High school	R	Car accident	B	T5	4
8	M	55	University	R	Spinal stroke	B	T5	2
9	M	51	University	R	Spinal stroke	B	T1	18
10	F	57	University	R	Bicycle accident	A	T6	3

Abbreviations: AIS, American Spinal Injury Association Impairment Scale [22]; LOI, level of injury as assessed by the International Standards for Neurological Classification of Spinal Cord Injury (ISNCSCI); SCI, spinal cord injury. High school = graduated from high school; University = undergraduate degree at least.

Ten control participants were included in the study, after being recruited through social networks and personal acquaintance with the researchers. To decrease variability, they were each matched to an SCI group participant with respect to age (±9 years; average difference 3.5 years), gender, hand dominance, and education (see Table 2). According to a dependent samples t-test, their mean age of 40.2 years (range: 21–59) did not differ significantly from that of the SCI group (*p* = .15).

**Table 2:** Control group: Demographic information.

Parti-cipant	Sex	Age (years)	Education	Dominant hand
1	M	47	High school	R
2	M	26	University	R
3	M	42	University	R
4	M	48	University	L
5	M	21	High school	R
6	M	25	High school	L
7	M	23	High school	R
8	M	56	University	R
9	F	59	University	R
10	M	55	University	R

High school = graduated from high school; University = undergraduate degree at least.

The study was approved by the Chaim Sheba Medical Center ethics committee. All participants entered voluntarily and signed a written informed consent form.

### Measures

#### International Standards for Neurological Classification of Spinal Cord Injury (ISNCSCI)

Severity of SCI was based on neurological level of injury (NLI; defined by lowest motor and sensory intact segment) and completeness or incompleteness of neurological damage, as defined by AIS grades A and B. AIS A indicates complete injury, with no sensory or motor function preserved in sacral segments S4-S5. AIS B indicates preservation of sensory but not motor function below the 'zone of injury' and includes sacral segments.

#### SRT task

During the task, participants were seated on wheelchairs adapted according to their neurological level of injury (e.g., high backrest for those with higher levels), facing a computer screen. Each was presented with a sequence of targets appearing in four positions on a screen and asked to press one of four keys corresponding to each position as quickly and precisely as possible. They were not informed that positions would appear in a repeated serial order. Reaction time changes over the course of the task served as a basis for assessing implicit learning of the series.

The design employed in the current study was based on the paradigm employed by Vakil and colleagues [25]. A red light appeared in one of four squares (3.3 x 3.3 cm) arranged horizontally on a 17" notebook computer screen. Participants were given the following instructions: "A red light will appear on one of the four positions on the screen. Using the index finger of your dominant hand, your task is to press as fast as possible one of the four horizontal buttons which are marked with a circle sticker on the key board that corresponds to the position of the red light.'' Before beginning the experiment, participants performed 12 training trials.

The red light position appeared in 12-trial sequences of repetitions (detailed below). Nine repetitions of a sequence (i.e., 108 trials) formed one block. Participants were presented with 8 blocks, with a 30 second rest between them. The first 6 blocks contained repetitions of the sequence 342312143241, with 1 as the left-most position and 4 the right-most. The seventh block contained repetitions of the sequence 341243142132, and the eighth block returned to the original sequence 342312143241. All sequences were adapted from previous research [26]. The sequences were second-level balanced: knowledge of the next position required knowledge of the two previous positions [27]. To discourage participants from recognizing the serial order of the sequence, the starting position was different in every block.

**Fig 1:**
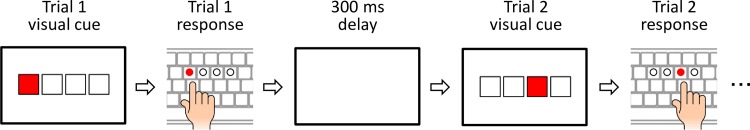
Schematic representation of the SRT task. In each trial, the visual cue appears and the participant responds by selecting the appropriate response on the keyboard. The cue then disappears, ending the trial and, following a fixed delay, another visual cue marks the beginning of a new trial. In the current study, there were 12 trials per sequence, 9 sequences per block, and 8 blocks, of which the first 6 used a repeating sequence, the 7^th^ used a random interference sequence, and the 8^th^ returned to the original repeating sequence (recovery).

Three hundred milliseconds after a response was made, or if the participant did not respond within 5 seconds, the next target appeared on the screen, whether or not the response made was correct. Reaction time was defined as the time from stimulus onset to response (key strike), and was recorded automatically by the computer for correct responses only. Incorrect responses were recorded as errors. A schematic representation of the task is shown in Fig 1.

The measures derived from the task included: *learning*, the reaction time decline in the first six blocks; *interference*, the incline in reaction time due to the series change (block 6 to 7); and *recovery*, the reaction time decline due to the change back to the learned sequence (block 7 to 8).

#### Generate task

This task was administered to enable comparison of the two groups in terms of explicit learning of the SRT task sequence. Following the eighth block, participants were informed that the positions in blocks one through six and eight had been presented in serial order. They were then presented with the first three positions of the original sequence and asked to continue it from memory by typing the correct positions on the keyboard. When the correct key was typed, a light turned on; when the wrong key was typed, nothing happened. For each position, participants continued trying until the correct response was entered, before moving on to the next position. Number of errors was calculated.

#### Depression

The Quick Inventory of Depressive Symptomatology Self-Report (QIDS-SR) [28] was employed to assess depression according to DSM-IV criteria. The questionnaire has been used previously to assess depressive symptoms following SCI [29,30] and its sensitivity is equal to self-report depression measures typically used in the past [31]. It contains 16 multiple choice questions with four answers each (0–3). Scores range from 0 (no depression) to 27 (major depression).

#### Additional cognitive measures

The Vocabulary and Matrices subtest of the Hebrew version (first edition) of the Wechsler Adult Intelligence Scale (WAIS-3) [32] were employed to estimate verbal and performance IQ, respectively. To assess auditory learning and memory, the Hebrew version of the Rey Auditory Verbal Learning Test (RAVLT) [33], a standardized, widely used, and valid measure of verbal memory function [34], was administered in the standard fashion [35]. The test involves memory for lists of 15 common words and yields free recall, delayed recall, and recognition measures before and after interference. Visual learning and memory were assessed using the Rey-Osterrieth Complex Figure Test (ROCFT) [36], in which participants are asked to copy a complex figure, to draw it from memory immediately following, and then again after 20 minutes. Scores were calculated according to standard form [37,38] and then normalized (M = 0, SD = 1).

### Procedure

Tests were administrated in one or two sessions. The SRT task was administrated between the RAVLT learning and delayed memory tasks. As noted above, the generate task was administrated immediately following the SRT task.

### Statistical Analysis

All statistics analyses were performed using IBM SPSS Statistics Professional 20.0, with a 0.05 level of significance.

For the SRT task, as the number of errors (i.e., incorrect responses) made by the participants was negligible, and all participants responded within the five-second time limit, the only dependent measure statistically analyzed was reaction time. As in previous studies [e.g., 19, 25], the median reaction time (RT) was calculated for every series of 12 items, forming 9 medians for each block, and the means of the medians for every block were analyzed. Initially, to examine the possibility of general motor speed deficits among the SCI participants, two-tailed paired sample *t*-tests were used to compare the reaction times of the control and experimental groups on the first block of the task. Then, three mixed-design repeated measures (RM) analyses of variance (ANOVAs) were performed with the between-subject variable group (SCI versus control) and the within-subject variable block, to assess the change in reaction time in the three measures derived from the task, as follows: learning (reaction time decline in blocks 1–6), interference (reaction time incline following series change, blocks 6–7), and recovery (reaction time decline following return to learned sequence, blocks 7–8).

Dependent sample *t*-tests were used to compare the number of errors made during the Generate task by the control and SCI groups.

The dependent measure for the RAVLT analyses was number of words remembered. Three mixed-design repeated measures ANOVAs were performed with the between-subject variable group (SCI versus control) and the within-subject variable trial, to assess the change in the number of words remembered in three measures derived from the task, as follows: learning (decline in trials 1–5), interference (incline following word list change, trials 5–6), and recovery (decline following return to first word list, trials 6–7).

Two-tailed dependent-sample *t*-tests were used to compare the control and SCI groups on scores derived from the QIDS-SR, the WAIS-3 subtests, and the ROCFT.

## Results

### SRT task

**Fig 2:**
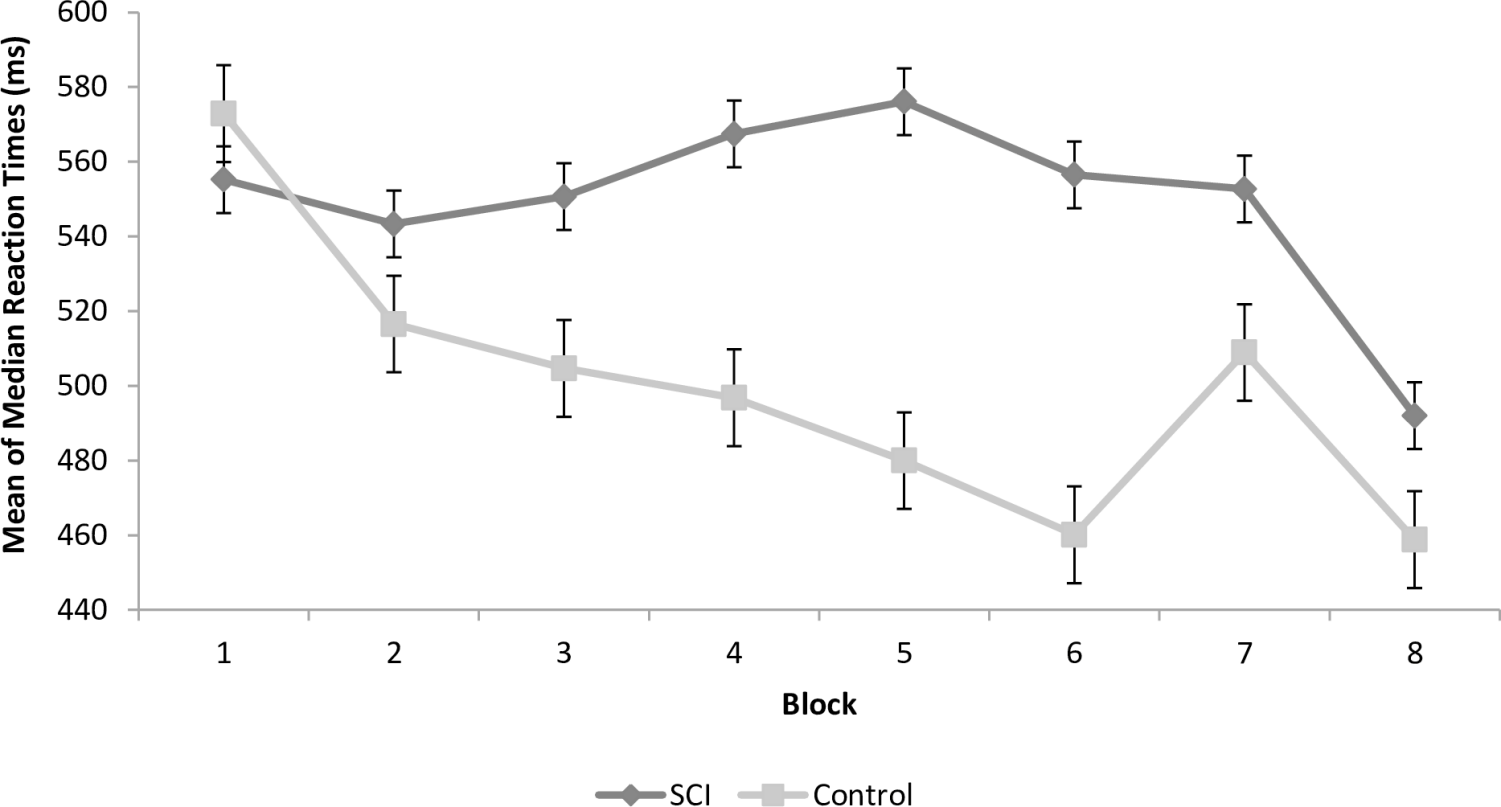
Serial Reaction Time (SRT) task performance (mean and SEM) in the spinal cord injury and control groups.

The results of the SRT task analyses (described above) are presented in Fig 2.

#### Baseline reaction time

No significant difference was found between the reaction times of the two groups on the first block of the SRT task (*p* = 0.62; Fig 2), indicating that potential differences between the learning curves of the two groups could not be attributed to an overall reduction in response speed in the SCI group.

#### Learning

There was a non-significant trend (*p* = 0.05) toward slower reaction times overall in the experimental group as compared to controls. The effect of block also approached significance (*p* = 0.07), with an overall decline in reaction times in the entire sample across blocks. These effects were qualified by the more relevant finding of a significant group by block interaction (*p* < 0.001). Specifically, as seen in Fig 2, follow-up analysis showed a learning effect in the control group (*p* < 0.001) that was absent in the SCI group (*p* = 0.73).

#### Interference

Overall, reaction time was faster in controls than in the SCI group (*p* < 0.05). No overall interference effect was found (*p* = 0.39). However, there was a significant group by block interaction (*p* < 0.05). Specifically, as seen in Fig 2, follow-up analysis showed an interference effect in the control group (*p* < 0.05) that was absent in the SCI group (*p* = 0.89). In the control group, reaction time was higher in the transition from block 6 to block 7. No such incline was found in the SCI group.

#### Recovery

In the transition from block 7 to 8, the control and SCI groups did not differ from one another (*p* = 0.27), and there was an overall decline in reaction time (*p* < 0.01). The group by block interaction was also significant (*p* < 0.05), as the reaction time decline in the transition from block 7 to block 8 was greater in the SCI group than in the control group (Fig 2).

### Generate Task

No group differences were found in number of errors made by the control (*M* = 8.00, *SD* = 2.24) and SCI groups (*M* = 6.90, *SD* = 3.45) in attempting to explicitly generate the original sequence (*p* = 0.18).

### RAVLT

#### Learning

**Fig 3:**
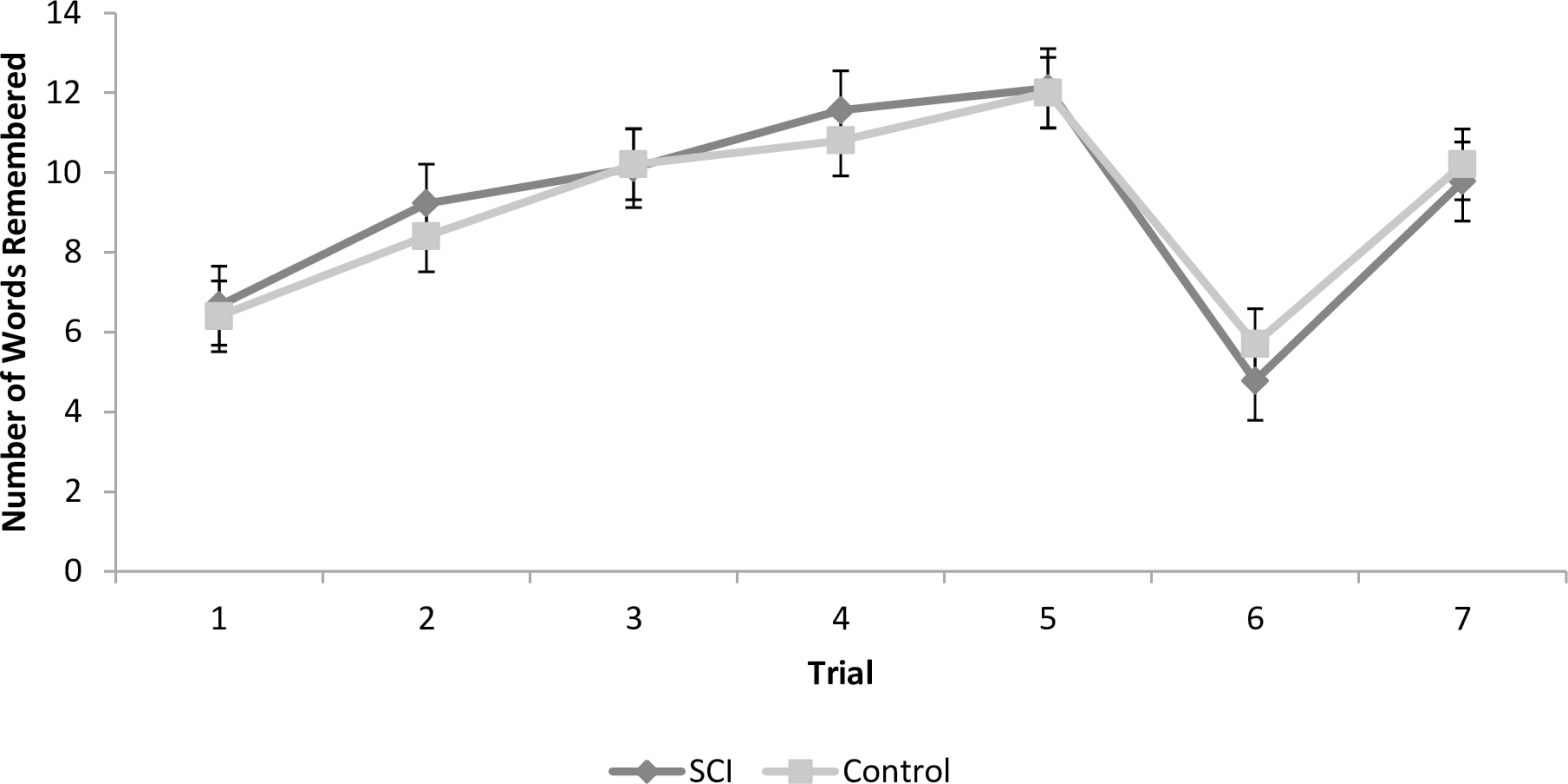
Rey Auditory Verbal Learning Task (RAVLT) performance (mean and SEM) in the spinal cord injury and control groups.

There was no difference between the groups in number of words remembered (*p* = 0.55). There was a learning effect over repetitions, indicating that both groups learned the words (*p* < 0.01), and no group by repetition interaction (*p* = 0.71; see Fig 3).

#### Interference

The control and SCI groups did not differ from one another (*p* = 0.54). An overall interference effect was found (*p* < 0.0001) with participants remembering significantly fewer words in the new word list trial. This pattern was found in both groups, such that there was no group by trial interaction (*p* = 0.23).

#### Recovery

In the transition from trials 6 to 7, the control and SCI groups did not differ from one another (*p* = 0.53), there was an overall decline in reaction time (*p* < 0.0001), and there was no group by trial interaction (*p* = 0.70).

Overall, the SCI and control groups showed no differences in performance on the RAVLT.

### QIDS-SR

There was no difference on the QIDS-SR measure between the SCI group, *M* = 7.11, *SD* = 4.65, and the control group, *M* = 4.60, *SD* = 2.68 (*p* = 0.29).

### WAIS-3 Subtests

There was no difference on the Vocabulary subtest between the SCI group, *M* = 8.67, *SD* = 3.32, and the control group, *M* = 10.80, *SD* = 1.32 (*p* = 0.09) or on the Matrices subtest, SCI group: *M* = 11.44, *SD* = 4.39, control group: *M* = 12.70, *SD* = 2.58 (*p* = 0.45).

### ROCFT

There was no difference on the delayed measure of the ROCFT between the SCI group, *M* = 0.15, *SD* = 1.30, and the control group, *M* = 0.67, *SD* = 1.03 (*p* = 0.34).

## Discussion

Research suggests that that there are fundamental differences between implicit learning and explicit learning, and that this has implications for the process of recovery after neurologic injury. Implicit learning is an unconscious, incidental acquisition of procedural knowledge. Explicit learning, in contrast, is a conscious, intentional, and declarative process of knowledge acquisition [39]. In the current study, distinct differences were found between the SCI and control groups in implicit procedural learning, which were not mirrored in measures of explicit learning and memory. Specifically, the two groups had similar baseline reaction times on the SRT task, ruling out the possibility that between-group differences could be attributed to response or processing speed alone. However, the SCI group showed no improvement in reaction time over the first six blocks and no advantage for the initially presented sequence as compared to the novel interference sequence presented in block seven. Thus, SCI participants deviated notably from the learning curve displayed by the control group, despite showing comparable results on both the generate task and the additional tests of declarative memory, learning, and intelligence. This clear cut dissociation represents the first empirical evidence of a specific deficit in implicit procedural learning following SCI, which may have broad implications for rehabilitation and adjustment.

While attesting to the specificity of the implicit learning deficit exhibited by SCI participants in the current study, the fact that the SCI group did not differ from the control group on any of the intelligence or declarative memory measures diverges from previous reports of SCI-related deficits in similar functions [1,40], adding to inconsistencies in the existing literature [5,41]. Such discrepancies likely arise from significant methodological differences between studies, involving injury characteristics, control groups, and assessment of depression, among other factors. However, the current finding that individuals with SCI can exhibit implicit learning deficits in the absence of a general cognitive decline indicates that examination of the specific mechanisms potentially underlying these deficits is warranted.

One possible mechanism by which cognitive deficits in SCI may be explained involves secondary changes in the brain. With respect to implicit learning, the basal ganglia are believed to be a region of interest [24,42,43], for example based on performance deficits in conditions in which this area is known to be damaged [44,45]. Increased activity in basal ganglia-thalamocortical pathways, which are involved in coordination between sensory input from the spinal cord and motor output [46], has been found in individuals with SCI during motor tasks using unaffected limbs [47] and while trying to move paralyzed limbs [48]. Taken together, these findings support the possibility that changes in this circuitry initiated by spinal cord damage may lead to altered performance on implicit or procedural learning tasks.

Depression, which occurs frequently following SCI [12], is another proposed source of cognitive deficits. Presumably, depressed mood following injury could cause a general decline that would be expressed in implicit learning, among other functions. In the current study, however, depression in the SCI group did not differ from that of controls, and there was no evidence of group differences on the additional cognitive measures. While this does not rule out the contribution of depression to cognitive deficits in SCI, it supports the existence of a direct relationship between SCI and impaired implicit motor sequence learning, not mediated by mood. This is in line with previous work [49] revealing no connection between cognitive functioning and depression in SCI.

It is also possible that cognitive deficits in SCI result from damage to spinal areas with a direct contribution to intact functioning. A recent groundbreaking fMRI study [17] showed evidence of local learning-induced plasticity in intact human spinal cord during motor sequence learning, suggesting that the spinal cord constitutes an active and distinct functional component of the human motor learning network. While the scope of the current study is not sufficient to determine the source of the observed deficit in motor sequence learning, the findings suggest that similar imaging studies examining damaged human spinal cord are warranted. Longitudinal studies following the performance of SCI patients on motor sequence learning tasks from immediately after the injury over time may also shed light on the mechanisms underlying implicit learning deficits in this population.

As noted above, there were no group differences in the generate task, showing that the groups did not differ from one another in terms of explicit learning of the same motor sequence examined in the SRT task. This is in line with the lack of group differences on the declarative memory components of the RAVLT and ROCFT, and should be considered within the context of well-based literature reporting that procedural and declarative learning and memory are often dissociable [18]. Indeed, the opposite pattern, in which procedural learning remains largely intact and explicit memory or learning is damaged, is believed to be prevalent following brain injury [50]. This distinction may be significant in tailoring rehabilitation programs to the needs of individuals with spinal injury both with and without brain injury. In this context, it may be useful to draw on rehabilitation methods used in Parkinson's disease, Huntington's disease, and frontal brain damage resulting in specific procedural learning deficits [51].

### Study limitations and directions for further research

The most notable limitation of the current study involved the small final sample size of ten participants, which was also heterogeneous in terms of cause of SCI and involved a broad range of time-since-injury. This resulted largely from our focus on screening for concomitant brain injury and potentially confounding premorbid conditions, as well as from the heterogeneity inherent in clinical samples of patients with SCI, in which interventions and manipulations must concur with treatment goals and schedules. Though controls were matched to SCI participants in an attempt to reduce variability, this has limited benefit, and future research should aim to examine implicit learning in larger or more homogeneous study samples. Apart from reducing the risk of type-II error, a larger sample size would make it possible to further examine potential effects of time-since-injury, such as recovery processes leading to improvement in procedural memory or, alternately, reduced neural feedback from the spinal cord to the brain leading to detrimental changes.

Further limitations stem from potential confounding factors such as long-term hospitalization and use of pain medications. These are characteristic of all research on clinical SCI samples. Given the interplay of premorbid, injury-related, and treatment-related factors, it is virtually impossible to find two SCI patients with identical profiles. Further, controlling for a small number of variables often leaves variability in others. In future research, some of this variability may be limited by comparing participants with SCI to individuals who sustained traumatic injuries not involving the spinal cord and had similar hospitalization and medication profiles. Also, it is likely that some variables, like depression, medication status, or fatigue, affect overall functioning rather than one specific task, and are thus unlikely to explain the distinct between-group differences on the SRT task.

Finally, while performance patterns on the SRT task differed dramatically between the control and SCI groups, with the latter showing virtually no decline in reaction time during the first six blocks, both groups showed a significant reaction time decline following the interference trial, which was even greater in the SCI group than in controls. This decline was to be expected for the control group, in which it presumably reflected the discrepancy between learned and unlearned sequences, but difficult to explain for the SCI group, in which the first sequence did not appear to be learned. Further research employing an SRT task with a greater number of blocks could potentially shed light on the source of this finding. It would also be useful in understanding the course and timing of motor sequence learning in individuals with SCI, and in determining whether they match the performance of controls over time.

## Conclusions

Compared with healthy control participants, individuals with SCI showed a dissociative pattern, with a specific deficit in implicit procedural learning that was not explained or accompanied by more general impairments in intelligence or memory. This novel finding is in line with clinical observations of difficulty in skill-learning following SCI and, though preliminary, may have important implications for rehabilitation. Most notably, the results suggest that individuals with SCI, like individuals with brain injury [52], may benefit from cognitive rehabilitation addressing their specific neuropsychological profiles, and from longer stays in rehabilitation wards to provide them with sufficient time and tailored training to acquire new skills.

## Supporting Information

S1 Dataset Raw data from serial reaction time (SRT) task, depression, and additional cognitive measures, for control and spinal cord injury (SCI) groups. (XLSX)
